# miR-379-5p affects breast cancer cell behavior by targeting UBE2E3 ubiquitin conjugating enzyme

**DOI:** 10.1371/journal.pone.0310315

**Published:** 2024-12-02

**Authors:** Araya K. Schroder, Conor J. Loy, Fernanda Aiala, Jazmyn Rafique, Arnob Ghosh, Lina I. Yoo

**Affiliations:** Department of Biology, Denison University, Granville, Ohio, United States of America; University of Delaware, UNITED STATES OF AMERICA

## Abstract

MicroRNAs (miRNAs) play an increasingly recognized role in modulating cancer development. Due to their function in regulating gene expression, miRNAs can suppress or promote tumorigenesis. miR-379-5p expression is downregulated in multiple human cancers, including breast and bladder cancers. However, the mRNAs targeted by miR-379-5p that promote cancer development have not been fully identified. Our goal was to identify a gene whose expression is regulated by miR-379-5p, and which may contribute to cancer development in cells where miR-379-5p expression is reduced. Bioinformatics analysis showed the UBE2E3 ubiquitin conjugating enzyme gene to be a potential target for miR-379-5p. To verify that UBE2E3 is a target, we transfected normal human epithelial mammary cells and breast adenocarcinoma cell lines with a miR-379-5p mimic. The mimic reduced UBE2E3 mRNA and protein levels, as would be predicted for a miR-379-5p target. To determine if UBE2E3 is a direct target of miR-379-5p, we engineered two luciferase reporter gene constructs to contain either a wild-type putative miR-379-5p binding sequence isolated from the 3’UTR of the UBE2E3 gene, or a scrambled sequence. The luciferase assay showed that the miR-379-5p mimic suppressed luciferase activity for the WT binding sequence reporter, but not for the scrambled reporter, showing that the effect of miR-379-5p on UBE2E3 expression is likely to be direct. Finally, to determine if the effect of miR-379-5p on UBE2E3 is related to cellular behaviors that play a role in cancer development, we measured cell viability by resazurin assay, cell proliferation by BrdU assay, and apoptosis by caspase 3/7 activation assay. The miR-379-5p mimic and silencing UBE2E3 expression both resulted in significantly diminished cell viability, while silencing UBE2E3 demonstrated both higher proliferation and apoptotic rates. Overall, these results suggest that while the overall effect of miR-379-5p is to inhibit breast cell viability and proliferation, the effect of silencing its target UBE2E3 is more complex because it induces both cell proliferation and apoptosis.

## Introduction

MicroRNAs (miRNAs) are short RNAs that have been shown to block protein expression of their target mRNAs [1]. There has therefore been much interest in developing miRNAs as therapeutic approaches to various human diseases caused by abnormal gene expression [2]. One disease in which there has been particular interest regarding the role of miRNAs is cancer [3]. Multiple miRNAs have been implicated as having oncogenic and tumor suppressive roles in cancer development.

We were interested in identifying microRNAs that are underexpressed in multiple cancer types according to data from the NCI Genomic Data Commons TCGA dataset. From our analysis, we were able to determine that miR-379-5p follows a pattern of reduced expression in breast, prostate, and bladder tumors. This finding was supported by previously published studies that found lower expression of miR-379-5p in breast [4], lung [5], endometrial [6], and bladder cancer [7] compared to normal tissues. We hypothesized that this could indicate an important tumor suppressive role for miR-379-5p, and that identifying the mRNA targets for this microRNA is crucial to understanding the mechanisms for how its reduced expression affects cells.

miR-379-5p is a microRNA that has been shown to have growth inhibitory effects in multiple kinds of cells including breast [4], endometrium [8], lung [5], intestine [9], bladder [7], oral squamous [10], and glioma [11]. Furthermore, some studies showed that miR-379-5p can promote apoptosis [5, 10]. The impact of miR-379-5p is likely to be tissue specific, however, as it has also been shown to promote the proliferation of chondrocytes [12, 13].

We set out to identify a novel mRNA target for miR-379-5p that we could verify through microRNA mimic overexpression studies, luciferase reporter assay, and cell function assays. Bioinformatics programs indicated UBE2E3 as a potential mRNA target for miR-379-5p. UBE2E3 is a ubiquitin conjugating E2 enzyme [14, 15]. Studies have shown that UBE2E3 promotes cell proliferation [16], and inhibits cellular senescence in retinal epithelial cells [17] and bone marrow mesenchymal stem cells [18]. We used breast cells grown in culture to investigate the previously untested hypothesis that UBE2E3 is a mRNA target for miR-379-5p.

## Materials and methods

### miRNA differential expression analysis

TCGA microRNA databases were accessed using the Genomic Data Commons (https://portal.gdc.cancer.gov/). Researchers did not have access to information that would allow identification of individual participants. Data was queried, downloaded, and aggregated using Python on May 25, 2018. miRNA with low normalized counts per million expression levels were removed prior to differential abundance analysis. Differential abundance analysis was performed on raw miRNA counts using the DESeq2 package in R. Data analysis and coding may be found here: https://doi.org/10.6084/m9.figshare.26069581

### Cell culture

AG11132 untransformed breast epithelial cells (NIA Aging Cell Culture Repository) were obtained from Coriell Institute of Medical Research and grown in MEGM medium (Lonza, CC-3150) supplemented with bovine pituitary extract, hEGF, insulin, hydrocortisone, and penicillin/streptomycin. MDA-MB-231 cells were obtained from the ATCC and grown in DMEM (Gibco), 10% fetal calf serum (Hyclone), and penicillin/streptomycin (Gibco). MCF cells were obtained from the ATCC and grown in EMEM (Hyclone), 10% fetal calf serum, and penicillin/streptomycin. All cells were maintained in a humidified 37C incubator with 5% CO_2_.

### Q-PCR

AG11132 cells were seeded into 60mm dishes. Cells were transfected with 67pmol of either miR-379-5p mimic (Sigma MISSION, HMI0547) or a negative control (Sigma MISSION, NCSTUD002) using RNAiMAX (Invitrogen) transfection reagent. After 48 hours, cells were harvested for RNA and qPCR was performed using the Azuraquant green one-step loRox kit (Azura Genomics). The primers used are in Table 1.

**Table 1:** Q-PCR primers.

Gene	Forward primer	Reverse primer
UBE2E3	5’ TGGAGTCCCGCTTTGACTAT-3’	5’ TGTCTGGCTATCCTGTCGTG-3’
B-actin	5’CACCATTGGCAATGAGCGGTTC-3’	5’AGGTCTTTGCGGATGTCCACGT-3’
HPRT	5’CATTATGCTGAGGATTTGGAAAGG-3’	5’CTTGAGCACACAGAGGGCTACA-3’

qPCR was performed in a MyGo Mini Real-Time Thermal cycler at 50°C for 10 minutes, followed by 95°C for 2 minutes, and then 40 cycles of 95°C for 5 seconds and 60°C for 30 seconds. Relative quantities of mRNA were calculated using the ΔΔCt method. Both B-actin and HPRT data were used as reference genes to calculate the geometric mean to normalize the UBE2E3 quantifications.

### Immunoblotting

Cells in 6 well clusters were transfected with 25pmol miR-379-5p mimic or the negative control using RNAiMAX. After 72 hours, cells were harvested for protein using RIPA buffer with protease inhibitor (Sigma, P8340). After sample buffer was added, samples were boiled and loaded on Bio-rad mini TGX gels and transferred to PVDF membranes using the TurboBlot system. Membranes were blocked in 3% milk dissolved in TBST. Primary antibodies used were against UBE2E3 (Invitrogen MA5-26364, mouse monoclonal, 1:2000) and tubulin (DSHB, 12G10, mouse monoclonal, 1:10,000). Secondary goat anti-mouse antibodies (Jackson Immunoresearch #115-036-003) used were conjugated to HRP and diluted in 3% milk/TBST (1:5000). Immobilon HRP substrate (MilliporeSigma) was used in the final step. All membranes were imaged and quantitated using an AlphaInnotech imager. Separate regions encompassing the UBE2E3 and tubulin bands were drawn. Background signal was subtracted from the regions, and the intensity ratios for UBE2E3:tubulin were determined.

### 3’UTR Reporter assay

Oligonucleotides were ordered from IDT, and the sequences are in Table 2.

**Table 2:** Oligonucleotides used in generation of 3’UTR reporters.

Gene	Oligonucleotide 1	Oligonucleotide 2
UBE2E3-3’UTR WT	5’CTAACTAGTTAGTCCTGAA**GTCTACCA**AATATTT -3’	5’CTAGAAATATTT**GGTAGAC**TTCAGGACTAACTAGTTAGAGCT -3’
UBE2E3-3’UTR mut	5’CTAACTAGTTAGTCCTGAA**TCAGTCAC**AATATTT -3’	5’CTAGAAATATT**GTGACTGA**TTCAGGACTAACTAGTTAGAGCT -3’

The oligonucleotide pairs were annealed for both the WT and mutant. The pMirGlo dual luciferase vector (Promega) was digested with SacI/XbaI, and the annealed oligonucleotides were ligated into the WT and mutant versions of the 3’UTR reporters.

MDA-MB-231 and MCF7 cells were seeded into 96 well white plates. The following day, cells were transfected with 100 ng per well of either the 3’UTR WT reporter or with the 3’UTR mutant reporter, plus 1 pmol per well of either the miR-379-5p mimic or the negative control miRNA using Lipofectamine 3000. After 48 hours a luminescence assay was performed using the Dual-Glo Luciferase assay (Promega) on a plate reader (Agilent Biotek), and the firefly luciferase activity was normalized to the Renilla luciferase activity.

### Resazurin assay

10^4^ MDA-MB-231 cells or 4 x 10^3^ AG11132 cells were seeded into wells in a 96 well plate. Three wells were given growth medium only to serve as a background control value in the absence of cells. The following day, cells were transfected with 1 pmol per well of miR-379-5p mimic, a negative control miRNA, siUBE2E3 (Sigma MISSION), or with miR-379-5p inhibitor (Sigma, HSTUD0547) using RNAiMAX. After 72 hours, cells and control wells were given Presto Blue resazurin (Invitrogen) and incubated at 37C for 1 hour. Afterwards, the 570 and 600nm absorbance in the wells was read using a plate reader (Agilent Biotek). The A_600_ was subtracted from each A_570_ value, and then the average control well value (from wells with no cells) was subtracted from each experimental value to correct for background.

### BrdU assay

3 x 10^3^ AG11132 cells were seeded in wells of a 96 well microplate. Cells were transfected with 1 pmol per well of either miR-379-5p mimic, a negative control RNA, siUBE2E3, or miR-379-5p inhibitor. 48 hours after transfection, BrdU from the BrdU cell proliferation assay kit (Cell Signaling) was added to the cells. After 24 hours incubation with the BrdU, cells were assayed for BrdU incorporation following the kit protocol. Absorbance was read at 450nm in a plate reader.

### Apoptosis assay

1.75 x 10^4^ MDA-MB-231 cells or 5 x 10^3^ AG11132 cells were plated into wells in a white plastic 96 well plate. The next day cells were transfected with 1 pmol per well of either a negative control miRNA, miR-379-5p mimic, or with siUBE2E3. After 72 hours, Caspase-Glo 3/7 reagent (Promega) was added to all wells, the plate was shaken for 30 seconds, and then the plate was incubated at room temperature for 1 hour. Luminescence readings were taken on the plate reader. The average luminescence reading from no cell control wells was subtracted from each reading from the wells with cells.

### Statistics

For miRNA differential expression analysis, p-values were calculated using a negative binomial distribution and adjusted for multiple hypothesis testing using the Benjamini-Hochberg method. One-way and two-way Analysis of Variance (ANOVAs) and two-tailed Welch’s T-tests were carried out using R Studio. *p*<0.05 was considered statistically significant.

## Results

### miRNA differential expression analysis and target identification

**Fig 1:**
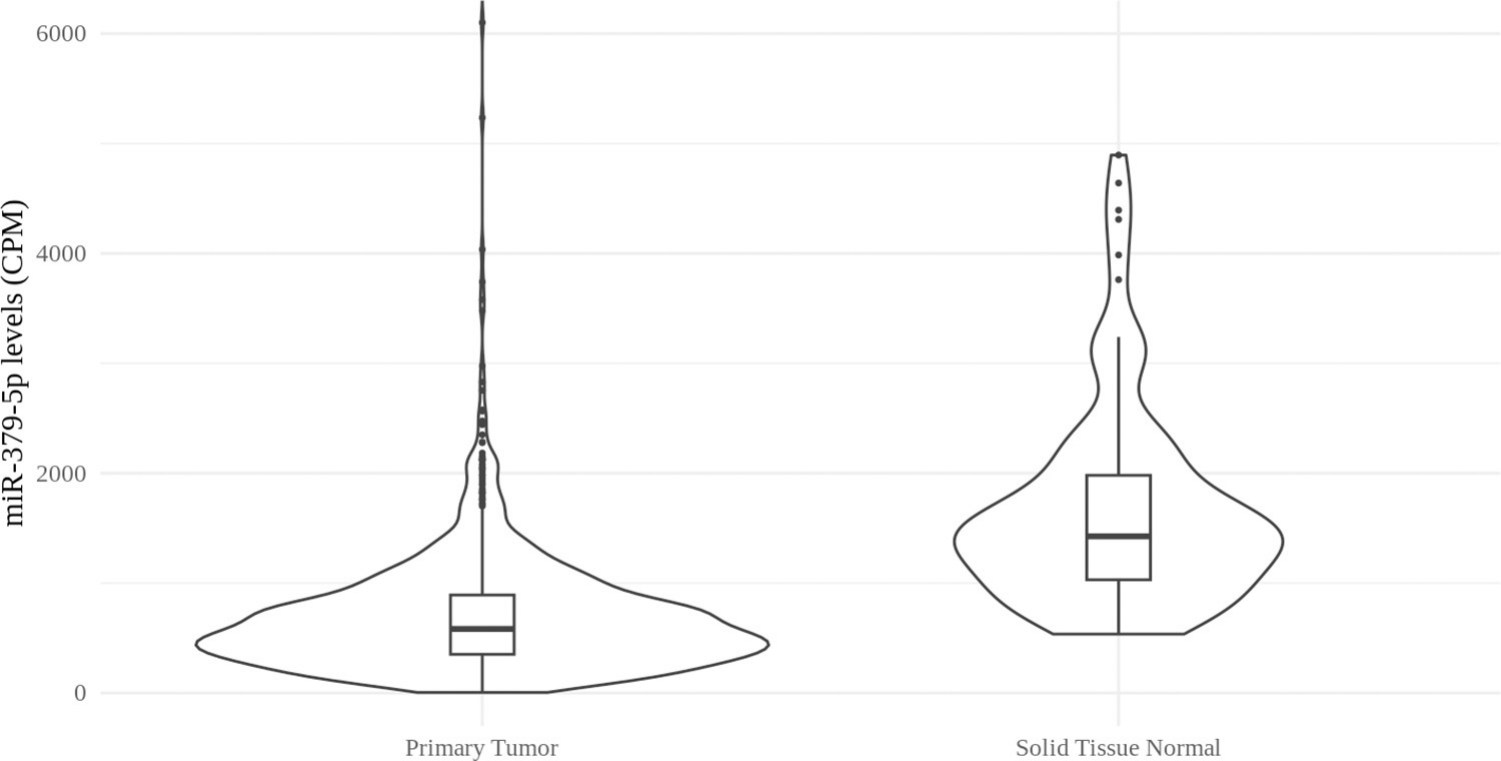
A miRNA differential expression analysis of miR-379-5p levels was done using TCGA data. 1096 primary breast cancer tumors and 104 normal solid tissue samples were used in this dataset. Boxplots indicate median and quartile values. Expression levels in normal compared to tumor samples were significantly different by Benjamini-Hochberg method, with an adjusted p-value of 7.14 x 10^−28^.

We were interested in identifying microRNAs that are expressed at lower levels in breast cancer cells. We carried out an analysis of miRNA expression using The Cancer Genome Atlas program miRNA-seq data from the Genomics Data Commons [19], which contained data from 1096 primary breast cancer tumors and 104 normal solid breast tissue samples. Average miR-379-5p levels were 2.2-fold lower in the breast tumors compared to normal breast tissue (FDR adjusted p-value 7.14 x 10^−28^) (Fig 1).

In order to identify possible mRNA targets for miR-379-5p, we used the miRDB online database (https://mirdb.org/) [20], TargetScan 8.0 (https://www.targetscan.org/vert_80/) [21], and PicTar (https://pictar.mdc-berlin.de/) [22]. The three predicted target genes that were ranked among the top 25 candidates on all three lists are shown in Table 3. All three bioinformatics prediction programs ranked UBE2E3 very highly as a potential target for human miR-379-5p, with TargetScan listing UBE2E3 as the 6^th^ ranked target, miRDB naming it as the 21^st^ ranked target, and PicTar assigning it a rank of 15^th^. UBE2E3 is a highly conserved E2 ubiquitin-conjugating enzyme, but very little is currently known about its function in eukaryotic cells.

**Table 3:** Highly ranked predicted hsa-miR-379-5p targets using multiple bioinformatic prediction tools.

Gene symbol	Gene name	TargetScan rank	miRDB rank	PicTar rank
UBE2E3	ubiquitin-conjugating enzyme E2 E3	6	21	15
ZBTB26	zinc finger and BTB domain containing 26	14	15	9
ELMOD2	ELMO/CED-12 domain containing 2	18	4	11

### Effect of miR-379-5p mimic on UBE2E3 expression in breast cells

**Fig 2:**
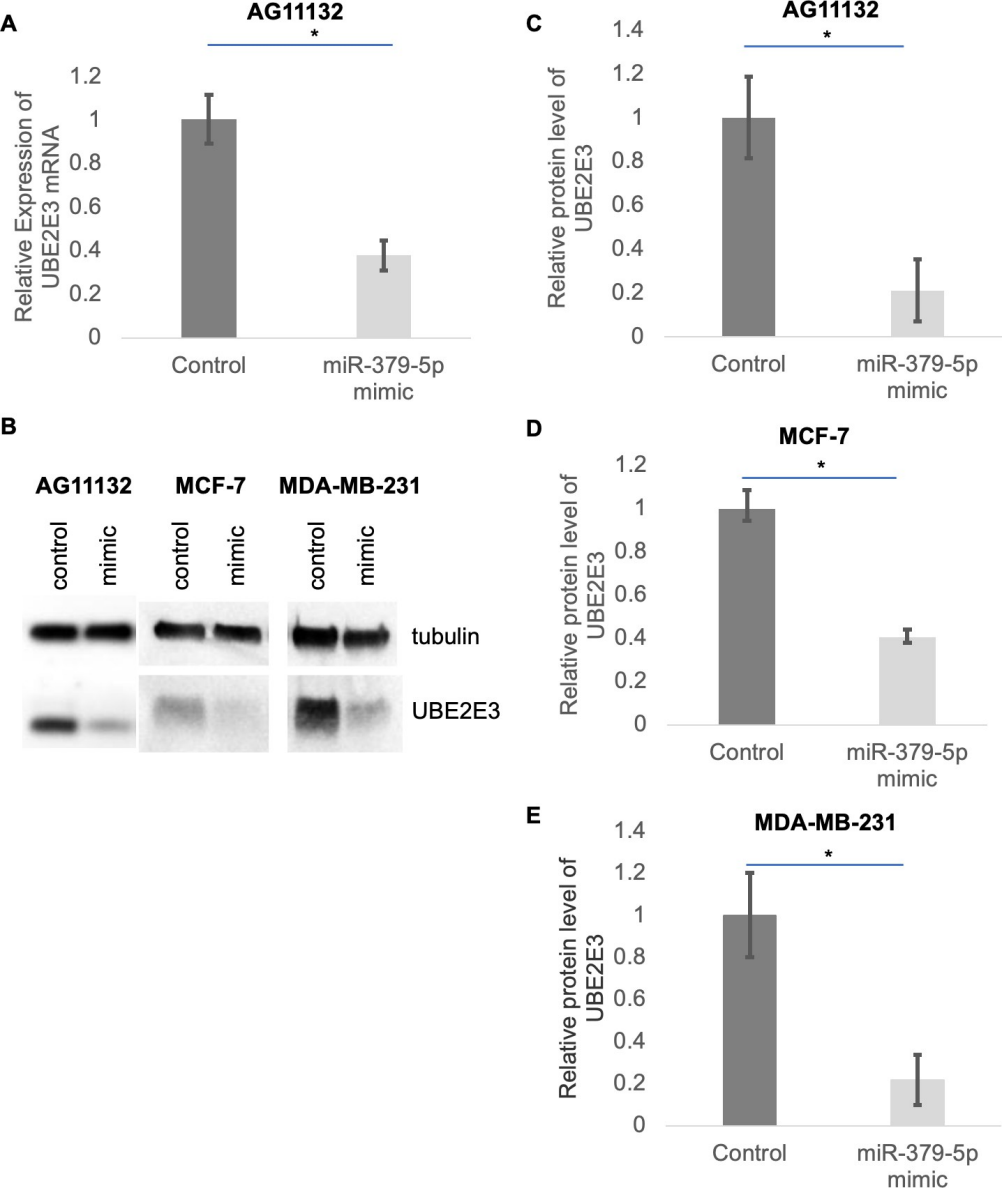
Effect of miR-379-5p mimic on UBE2E3 expression in breast cells. (A) UBE2E3 mRNA was measured by qPCR in AG11132 normal breast cells transfected with a negative control or miR-379-5p mimic. (B) UBE2E3 protein levels were measured by immunoblot in AG11132 cells, and breast adenocarcinoma cell lines MCF7 and MDA-MB-231. (C-E) Quantifications of immunoblots, where the average UBE2E3 expression level normalized to tubulin in cells transfected with the negative control was set to 1. All bars are means +/- standard deviations. All experiments were performed three times. *Welch’s t-test, p<0.01.

AG11132 human breast cells from a reduction mammoplasty were transfected with either a miR-379-5p mimic or a negative control miRNA. After 48 hours, the RNA was harvested from the cells and reverse transcription quantitative PCR was performed to measure levels of UBE2E3 mRNA. We found a significant decrease in UBE2E3 mRNA levels in the cells that had been transfected with the miR-379-5p mimic (Fig 2A), suggesting that the miRNA was causing UBE2E3 mRNA degradation. Next, we examined effects of the miR-379-5p mimic on UBE2E3 protein levels in AG11132 cells, as well as MCF7 and MDA-MB-231 breast adenocarcinoma cell lines (Fig 2B–2E). In all three cell types, the mimic induced a significant decrease in UBE2E3 protein levels, which was consistent with what was occurring to the UBE2E3 mRNA.

### UBE2E3 is a direct target of miR-379-5p

**Fig 3:**
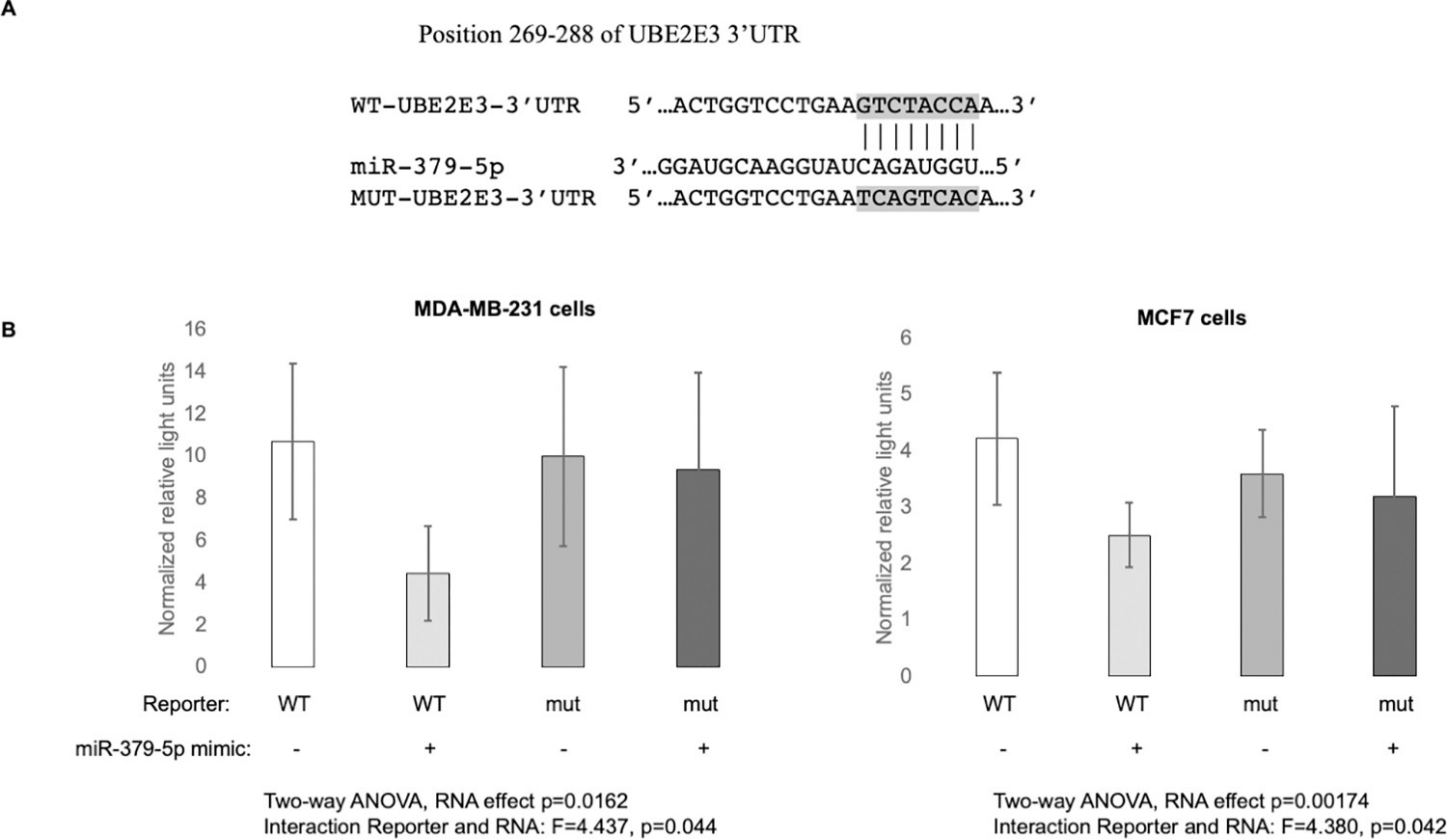
3’UTR Reporter assay to identify UBE2E3 as a direct target of miR-379-5p. (A) Alignment of UBE2E3 3’UTR and miR-379-5p. The seed position is at 281–288 in the 3’UTR. Sequence of mutant 3’UTR reporter is shown. (B) Luciferase activity using the reporters in the presence of control or miR-379-5p mimic in MDA-MB-231 and MCF7 cells. Two-way ANOVA was used for the analysis. All bars are means +/- standard deviation. Both experiments were carried out three times.

In order to address the possibility that the effect of miR-379-5p on UBE2E3 is indirect, we engineered a dual luciferase reporter construct with either the putative miR-379-5p binding site from the 3’UTR of the UBE2E3 gene, or with a scrambled binding site incorporated downstream of the firefly luciferase gene (Fig 3A). The reporter constructs were cotransfected into MDA-MB-231 and MCF7 cells (Fig 3B) with either negative control miRNA, or with the miR-379-5p mimic. We used a two-way ANOVA to examine the effect of reporter type and miRNA type on luciferase activity. The luminescence assay showed that the miR-379-5p mimic caused a significant reduction in light production from the reporter with the wild-type UBE2E3 3’UTR sequence, whereas the miR-379-5p mimic had an insignificant effect on light production from the mutant reporter. The interaction between reporter type and miRNA type on luciferase activity was significant (p<0.05). This provided evidence that miR-379-5p directly binds to the 3’UTR of the UBE2E3 mRNA to target it for degradation.

### miR-379-5p and UBE2E3 have opposing effects on breast cell viability

**Fig 4:**
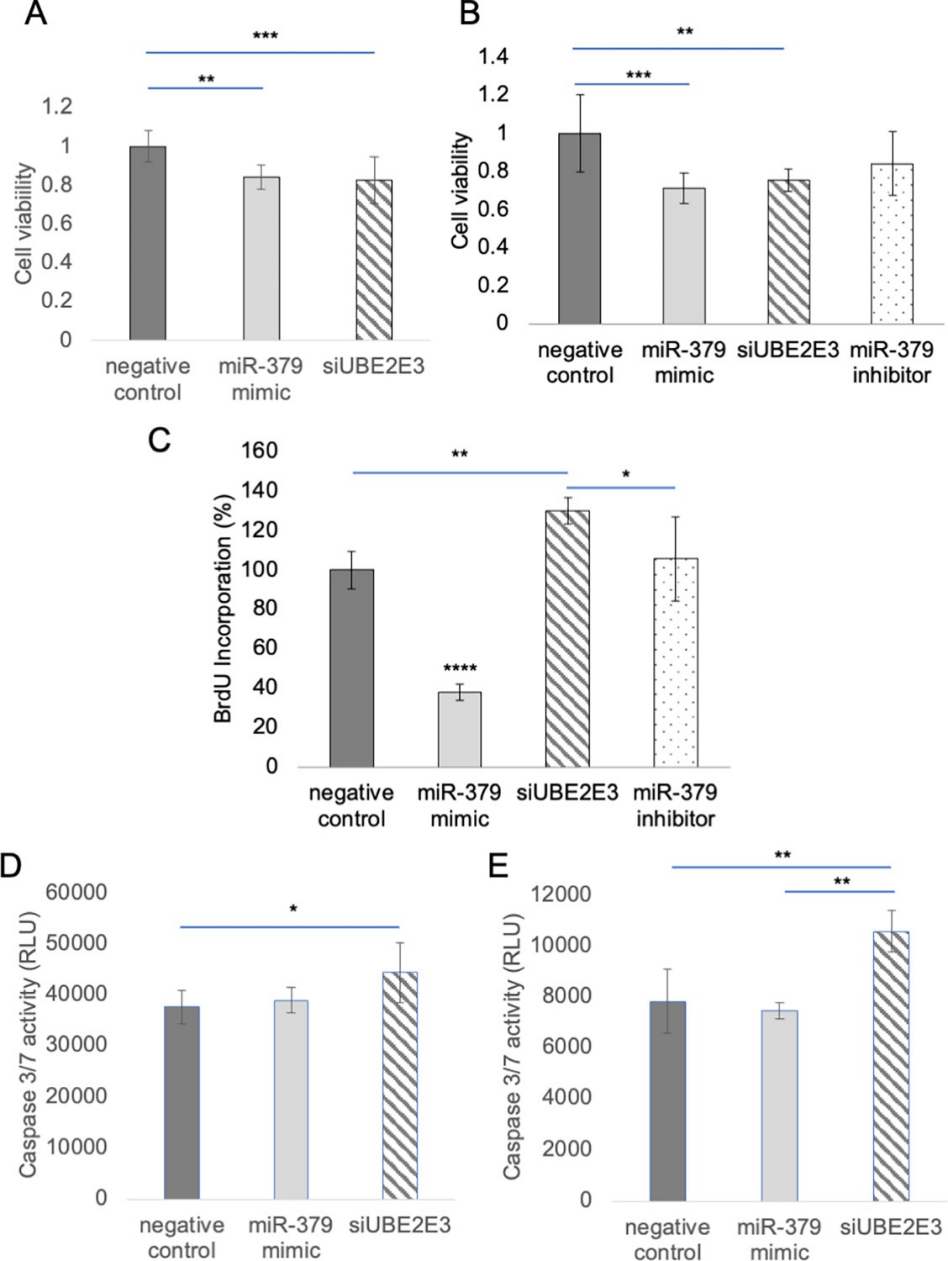
Effect of silencing UBE2E3 on cell viability and apoptosis. (A) PrestoBlue resazurin cell viability assay was used in transfected MDA-MB-231 cells. After 72 hours, PrestoBlue was added to the wells. One-way ANOVA p = 0.000268. Tukey-Kramer **p<0.01; ***p<0.001. (B) PrestoBlue cell viability assay in transfected AG11132 normal breast cells. One-way ANOVA p = 0.000274. (C) BrdU cell proliferation assay in AG11132 cells. One-way ANOVA p = 3.28e-10. Tukey-Kramer *p<0.05; ****p<1e-6 compared to all other treatments. (D) Caspase 3/7 activity assay in MDA-MB-231 cells 72 hours after transfection. One-way ANOVA, p = 0.0156. All bars are means +/- standard deviation. (E) Caspase 3/7 activity in AG11132 cells. One-way ANOVA p = 0.0005.

Previous studies have shown that miR-379-5p mimics inhibit MDA-MB-231 cell proliferation [4]. We decided to see if silencing UBE2E3 expression would have an effect on cell viability levels, since our study indicated that the miR-379-5p mimic diminishes UBE2E3 expression. We transfected MDA-MB-231 breast cancer cells and AG11132 normal breast cells with either a negative control miRNA, the miR-379-5p mimic, or with siUBE2E3. After 72 hours, the cells were measured for cell viability using a PrestoBlue resazurin assay. In both cell types, the cells that were given the miR-379-5p mimic or the siUBE2E3 had significantly lower cell viability than the control cells (Fig 4A and 4B). This showed that UBE2E3 promotes cell viability, and suggests that suppression of UBE2E3 expression by miR-379-5p may restrain breast cancer cell numbers. Notably, we also transfected the AG11132 cells with a miR-379-5p inhibitor (Fig 4B), but the inhibitor did not have a significant effect on cell viability. We have not quantified the amount of endogenous miR-379-5p in the AG11132 cells; it is possible that the inhibitor has no effect due to low levels of endogenous miR-379-5p in this case.

### miR-379-5p and UBE2E3 inhibit cell proliferation

The resazurin cell viability assay does not directly measure cell proliferation, so we performed a BrdU assay to determine the effect of miR-379-5p and UBE2E3 on MDA-MB-231 cell proliferation. We transfected the cells with a negative control, miR-379-5p mimic, siUBE2E3, or a miR-379-5p inhibitor. As expected, the miR-379-5p mimic caused a very significant decrease in BrdU incorporation in cells compared to the control (Fig 4C). Surprisingly, siUBE2E3 caused a significant increase in BrdU incorporation compared to the control. This result was unexpected given our previous finding that siUBE2E3 decreases cell viability by resazurin assay (Fig 4A).

### UBE2E3 inhibits apoptosis

The findings that siUBE2E3 increases cell proliferation but also decreases cell viability presented the possibility that silencing UBE2E3 may promote cell death. To test this idea, we transfected both the MDA-MB-231 cells and AG11132 cells with either the negative control miRNA, the miR-379-5p mimic, or with siUBE2E3. After 72 hours, the cells were tested for caspase 3/7 activation. We found that there were significantly higher levels of apoptosis in the cells in which UBE2E3 had been silenced (Fig 4D and 4E), suggesting that UBE2E3 promotes cell survival. Surprisingly, the miR-379-5p mimic did not induce apoptotic activity, unlike the siUBE2E3. It is possible that miR-379-5p targets other than UBE2E3 counteract the apoptotic induction due to UBE2E3 silencing. Alternatively, since siUBE2E3 more thoroughly silences UBE2E3 expression than the miR-379-5p mimic (data not shown), it is possible that the higher level of silencing of UBE2E3 is needed to see this effect on apoptosis.

## Discussion

There is a growing body of evidence that miR-379-5p is expressed at lower levels in a wide variety of human tumors, and consistent with these findings, miR-379-5p suppresses growth of cells in culture. To illuminate the mechanism by which miR-379-5p does this, we used bioinformatics microRNA target prediction programs and identified UBE2E3 as a possible mRNA target for miR-379-5p. Our studies showed that miR-379-5p mimics reduce UBE2E3 expression at both the mRNA and protein level in normal breast cells as well as breast cancer cell lines. By engineering the putative miR-379-5p binding site from the 3’UTR of the UBE2E3 gene into a luciferase reporter, we found that miR-379-5p is likely to directly bind to UBE2E3 mRNA. In addition, by silencing UBE2E3, we were able to determine that UBE2E3 promotes cell viability and survival in breast cancer cells. We have not seen any other previously published papers that have linked UBE2E3 silencing with increased apoptosis rates in cells. While miR-379-5p mimic alone does not appear to affect apoptosis rates in cells in our hands, other target genes for miR-379-5p have been identified, including EIF4G2 [9, 23], ROR1 [6, 8, 10], IGF1 [24, 25], PDK1 [26, 27], and FAK [28, 29]. It is possible that these other genes may mitigate the effects of UBE2E3 silencing on apoptosis rates. Altogether, our studies suggest that UBE2E3 is a direct target of miR-379-5p, and the intriguing possibility that reduced expression of miR-379-5p in many cancers may promote cancer development through the lost suppression of UBE2E3 expression and increased cell survival is worthy of future investigation. We will note here that a correlation analysis of miR-379 and UBE2E3 RNAseq gene expression levels in TCGA Breast Cancer (BRCA) samples using the UCSC Xena platform does not reveal any significant correlation between the two [30]. miR-379-5p may not be the only determinant of UBE2E3 expression levels. However, our results show that miR-379-5p does suppress UBE2E3 expression in breast cells, and will thereby affect breast cell viability.

UBE2E3 is a highly conserved E2 ubiquitin conjugating enzyme that appears to be restricted to monoubiquitylation of its targets [31]. A number of studies have been published in the last few years elucidating some possible targets and pathways that are impacted by UBE2E3. Our study is the first one to show that UBE2E3 can suppress cell proliferation in cells. An earlier study had shown that UBE2E3 can promote cell proliferation through its interaction with activated E3 ubiquitin ligase Cbl [32], resulting in increased protein stability for EGFR. Silencing UBE2E3 has also been shown to cause an increase in the cyclin-dependent kinase inhibitor p27, leading to a cell cycle exit and increase in cell size in retinal pigment epithelial cells [16].

In addition, UBE2E3 also appears to inhibit the development of cellular senescence. Plafker et al. [17] showed that blocking UBE2E3 expression results in senescence induction by boosting expression levels of p53, p21, and p16. Silencing UBE2E3 also results in relocation of Nrf2 away from the nucleus [18, 33], reducing Nrf2’s ability to inhibit cellular senescence [34]. The regulation of senescence by Nrf2 is closely tied to its induction of proteins that promote redox homeostasis in the cell. Oxidative stress is known to induce autophagy [35], and studies have shown increased basal autophagic flux in cells with silenced UBE2E3 [17]. Interestingly, UBE2E3 was also found to interact with Mulan E3 ubiquitin ligase and GABARAP, a member of the LC3 family that plays an important role in autophagy induction [36].

The studies in this paper have shown how reduction in miR-379-5p expression in tumors and increase in UBE2E3 expression may cooperate to promote tumor development. One other microRNA, miR-143-3p, has been found to target UBE2E3 as well [37–39]. We suggest that future studies are needed to fully elucidate regulation of UBE2E3, as well as identification of UBE2E3 targets and how UBE2E3-mediated ubiquitylation affects their function. Furthermore, the mechanisms by which UBE2E3 regulates cell viability remain to be fully understood. Ultimately, in vivo studies to assess the potential for miR-379-5p mimics in murine tumor models may present a novel therapeutic approach for cancer.
